# Correspondence

**Published:** 1987-08

**Authors:** T. J. David

**Affiliations:** *Dept of Child Health* University of Manchester, Booth Hall Children's Hospital, Manchester M9 2AA


Thank you for giving me the opportunity of replying to Dr. Hamlyn's letter.

Allergic reactions have been recognised from ancient times. In the 1920s and 1930s a fashion developed for incriminating food allergy in a large number of hitherto unexplained phenomena (1). The uncritical and overenthusiastic nature of the claims, plus the very anecdotal evidence upon which they were based, generally discredited the subject of allergy. Indeed the field of food allergy at that time has been described as "a model of obstruction to the advancement of learning" (1). The need for objective evidence became clear, and in the succeeding decades the study of allergy has increasingly acquired a scientific footing. Nevertheless, it is quite fair to say that a proportion of the medical profession is indeed "allergic to allergies". This is partly attributable to ignorance, partly to the lack of objective evidence for certain overenthusiastic claims (eg allergy to members of one's family, or to North Sea Gas, or environmental pollution causing a "runaway epidemic" of allergic disease), and partly to the extreme activities of many private allergy clinics. However the neglect of allergy by one section of the profession can hardly be taken as an excuse for the bogus activities of others.

It is clear that many subjects presenting with "allergy" do not have allergic disease at all (2, 3). The private allergist is often depicted as nothing more than a friendly neighbourhood crank. The reader may decide whether the word "crank" is appropriate in the context of failing to diagnose and treat important disease, providing unproven or bogus methods of investigation and treatment, inventing non-existent disease, and relieving anxious and gullible and often very hard-up parents of many hundreds of pounds. The connection between certain allergy clinics and scientology is a further matter of concern. The epidemic of refugees from private allergy clinics is stretching our own resources, and the casualties are not always treatable. To say in defence of bogus allergy treatment that by chance it sometimes does good is, in the present context, akin to saying that even though thalidomide does cause the occasional problem it is really quite a good sedative.

**REFERENCES**

1. MAY C. D. (1982) Food allergy: lessons from the past. *J Allergy Clin Immunol* **69**, 255–259.
2. PEARSON D. J., RIX K. J. B., BENTLEY S. J. (1983). Food allergy: how much in the mind? A clinical and psychiatric study of suspected food hypersensitivity. *Lancet* **1**, 1259–1261.
3. RIX K. J. B., PEARSON D. J., BENTLEY S. J. (1984) A psychiatric study of patients with supposed food allergy. *Brit J Psychiat* **145**, 121–126.

T. J. David. PhD, MD, FRCP, DCH.
*Dept of Child Health*
University of Manchester,
Booth Hall Children's Hospital,
Manchester M9 2AA.

